# Role of Autophagy on Heavy Metal-Induced Renal Damage and the Protective Effects of Curcumin in Autophagy and Kidney Preservation

**DOI:** 10.3390/medicina55070360

**Published:** 2019-07-10

**Authors:** Sabino Hazael Avila-Rojas, Alejandra Lira-León, Omar Emiliano Aparicio-Trejo, Laura María Reyes-Fermín, José Pedraza-Chaverri

**Affiliations:** Department of Biology, Faculty of Chemistry, National Autonomous University of Mexico (UNAM), Mexico City 04510, Mexico

**Keywords:** heavy metal, autophagy, curcumin, kidney, autophagosome fusion

## Abstract

Curcumin is a hydrophobic polyphenol compound extracted from the rhizome of turmeric. The protective effect of curcumin on kidney damage in multiple experimental models has been widely described. Its protective effect is mainly associated with its antioxidant and anti-inflammatory properties, as well as with mitochondrial function maintenance. On the other hand, occupational or environmental exposure to heavy metals is a serious public health problem. For a long time, heavy metals-induced nephrotoxicity was mainly associated with reactive oxygen species overproduction and loss of endogenous antioxidant activity. However, recent studies have shown that in addition to oxidative stress, heavy metals also suppress the autophagy flux, enhancing cell damage. Thus, natural compounds with the ability to modulate and restore autophagy flux represent a promising new therapeutic strategy. Furthermore, it has been reported in other renal damage models that curcumin’s nephroprotective effects are related to its ability to regulate autophagic flow. The data indicate that curcumin modulates autophagy by classic signaling pathways (suppression of protein kinase B (Akt)/mammalian target of rapamycin (mTOR) and/or by stimulation of adenosine monophosphate-activated protein kinase (AMPK) and extracellular signal-dependent kinase (ERK) pathways). Moreover, it allows lysosomal function preservation, which is crucial for the later stage of autophagy. However, future studies of autophagy modulation by curcumin in heavy metals-induced autophagy flux impairment are still needed.

## 1. Introduction

Curcumin or diferuloylmethane (1,7-bis(4-hydroxy-3-methoxyphenyl)-16-heptadiene-3,5-dione) is a hydrophobic polyphenol extracted from the rhizome of *Curcuma longa* L., also known as turmeric [1], which is widely grown in the southern and south western tropical regions of Asia [2]. Curcumin is formed by two aromatic rings (with o-methoxy phenolic groups) linked to an α,β-unsaturated β-diketone moiety [3]. Curcumin can exist in tautomeric forms, keto and enol [4]. It has been widely used in traditional medicine due to its multiple antioxidant [5], antitumor [6], antiviral [7] and anti-inflammatory properties [8], among others [1,3]. Furthermore, studies have shown its protective effect in multiple diseases such as cancer [6], and neurological [9], metabolic [10], lung [11], liver [5], renal [12], and cardiovascular diseases [2]. In addition to its many therapeutic activities, curcumin has the advantage of its biosafety in animals as well in healthy humans, even at high doses (12 g per day), without undesirable effects [13]. In addition, it has been shown that curcumin provides a nephroprotective effect against various xenobiotics, including heavy metals [12,14,15,16,17].

Heavy metals comprise elements like cadmium (Cd), lead (Pb), arsenic (As), mercury (Hg), and chromium (Cr), which are located along the Earth’s crust in various forms [18]. These elements are widely used in different anthropogenic activities, ranging from agriculture to industry [19]. Heavy metals can enter to the body by three routes: Intake (contaminated water or food), inhalation, and dermal contact [20]. Prolonged exposure (occupational or environmental) can cause serious problems in human health, especially in the kidneys, since they represent the major route of heavy metal excretion from the body [18]. Adverse effects of heavy metals have been usually associated with oxidative stress, which involves an overproduction of reactive oxygen species (ROS) and the loss of the endogenous antioxidant defense, as well as with mitochondrial dysfunction [18,21]. However, it was recently found that heavy metals can also impair autophagy flux [22,23,24], an evolutionarily-conserved self-digestive process, which is generally considered to be a cell survival mechanism [25]. In vitro and in vivo studies related to the participation of autophagy in kidney damage have described that autophagy helps to remove damaged mitochondria, avoiding ROS overproduction and cellular damage [26,27].

In this review we summarized the mechanisms involved in autophagy regulation by curcumin, as well heavy metals-induced autophagy flux impairment in the kidney. On the other hand, curcumin administration has been able to restore renal function in several models of kidney damage [8,14,28,29]. Furthermore, in hyperoxaluria, maleate and contrast-induced nephropathy, curcumin’s nephroprotection has been related to its ability to modulate the autophagy flux [17,30,31]. However, curcumin’s effect on heavy metals-induced autophagy flux impairment has not been explored yet, generating the opportunity for exploration in future studies.

## 2. Curcumin’s Antioxidant Effects and Mitochondrial Protection in Kidney Damage Models

Curcumin’s direct antioxidant effect derives from the presence of conjugated double bonds in its structure, allowing curcumin to act as an electron donor [32]. Therefore, it scavenges superoxide anion (O_2_^●−^), hydroxyl radical (^●^OH), singlet oxygen (^1^O_2_), hydrogen peroxide (H_2_O_2_), nitric oxide (NO^●^), peroxynitrite (ONOO^−^), and peroxyl radical [33]. Moreover, curcumin also activates the antioxidant response element (ARE) by the kelch-like ECH-associating protein (Keap1)/nuclear factor erythroid 2-related factor 2 (Nrf2) system [34]. The protective effect of curcumin has been described in kidney damage models induced by ischemia/reperfusion [8], cisplatin [35], 5/6 nephrectomy [16], and heavy metals (Pb, Cd, Cr) [12,14,15], where its antioxidant activity is highlighted. In addition, it also preserves mitochondrial function [16,29,35], which also contributes to renal function preservation in kidney function. The anti-inflammatory effect of curcumin also plays a prominent role in acute and chronic kidney damage models [36,37,38]. In this regard, curcumin has an anti-fibrotic effect in glomerulonephritis model, which involves the reduction of transforming growth factor-β1 and fibronectin production [36]. This effect is dependent on induction of heme oxygenase-1 (HO-1), a target of Nrf2. Following the same route, Ghosh et al. [37] in the 5/6 nephrectomy model, highlighted the effect of curcumin on the development of chronic renal failure, where inflammation plays an important role through tumor necrosis factor alpha (TNFα) and the transcription factor nuclear kappa B (NF-κB). In this model, curcumin partially suppressed the TNFα-mediated NF-κB activity and avoided macrophage infiltration, as well as the functional (such as proteinuria, blood urea nitrogen, and plasma creatinine) and structural (tubular atrophy, hyperplasia, and glomerulosclerosis) alterations. Furthermore, in the renal ischemia/reperfusion model, curcumin attenuated interferon gamma (IFNγ) expression, while increasing the IL-10 levels [38]. On the other hand, in an in vitro and in vivo model of rhabdomyolysis, curcumin reduced renal damage associated with rhabdomyolysis. In addition to its antioxidant and anti-inflammatory effect, curcumin decreased ferroptosis-mediated cell death [39]. Interestingly, the inhibition of this type of autophagy associated to iron metabolism, is able to attenuate the function and structural alterations in the kidney. Finally, the protective effect of curcumin was also mediated by HO-1 [39].

## 3. Autophagy and Its Evaluation

Autophagy is a biological process that allows the preservation of cellular homeostasis by the removal of damaged macromolecules and/or organelles in response to a variety of stimuli [40]. Autophagy consists of five steps: (1) Formation of the phagophore, a complex of Beclin-1, phosphatidylinositol 3-kinase/vacuolar protein sorting 34 (VPS34) and VPS15, (2) phagophore elongation and cargo recruitment, (3) autophagosome maturation, (4) fusion between the autophagosome and lysosome, and (5) autolysosome degradation [41,42]. In addition, formation of autophagosomes and autolysosomal degradation are essential stages to evaluate if the autophagy flux is functional. The usual hallmarks evaluated in these steps are the levels of: Microtubule-associated protein 1 light chain 3 (LC3-I) and its phosphatidyethanolamine form (LC3-II), as well as the ubiquitin binding protein p62, also called sequestosome 1. The LC3-II is an essential protein for elongation and closure of the phagophore, localized in the autophagosome membranes. Meanwhile, p62 is a receptor protein, which binds by its ubiquitin domain to the specific cargo, to anchor them to the LC3-II, present inside the autophagosome, to the subsequent cargo’s degradation in autophagolysosomes [43,44].

In relation to the signaling pathway, key regulators of autophagy include the class I phosphatidylinositol 3-kinase (PI3K) and adenosine monophosphate-activated protein kinase (AMPK) and the autophagy inhibitor the mammalian target of rapamycin (mTOR) [34]. mTOR activation is associated to the PI3K/protein kinase B (AKT)/p70 ribosomal protein S6 kinase (p70S6K) pathway and linked to growth factors. Thus, mTOR negatively regulates autophagy. Meanwhile, the AMPK kinase senses intracellular adenosine triphosphate levels and inhibits formation of the multiprotein kinase complex 1 of mTOR (mTORC1), leading to its dissociation from the Atg2/unc-51-like kinases complex (ULK) and to the dephosphorylation/activation of ULK1 and/ or ULK2, which triggers autophagy’s initiation [45]. Likewise, mTORC1 is also regulated by tuberous sclerosis complex 1 (TSC1)/TSC2 and acts as a brake for this pathway, therefore modulating autophagy [42]. In this regard, Akt phosphorylates TSC2 and inactivates the TSC1/TSC2 complex, meanwhile its phosphorylation by AMPK has the opposite effect [42].

## 4. The Autophagy in Heavy Metals Kidney Damage

The role of autophagy in renal injury is still under debate [46]. It has been described in tubular epithelial cells that autophagy acts as a survival mechanism in multiple renal damage models, including cisplatin, cyclosporine, and ischemia [47]. However, its overactivation can be counterproductive, leading to autophagic cell death [48]. Therefore, due to the dual role of autophagy, factors such as the temporality and intensity of the stimulus have to be considered a priori, to determine the role of this process.

In relation to heavy metal-nephrotoxicity (as summarized in Table 1), the participation of autophagy in cadmium chloride (CdCl_2_)-induced damage in mice has been demonstrated [49]. It was shown that high doses of CdCl_2_ increase the LC3-II/LC3-I ratio and promote the formation of autophagosomes, which lead to autophagic cell death, instead of mitigating the renal damage [49]. This is consistent with the study of Shi et al. (2019), which revealed that prolonged exposure to CdCl_2_ increased apoptosis in chicken kidneys by c-Jun N-terminal kinase (JNK)-dependent autophagy [50]. In the same sense, Liu et al. (2017) found in rat proximal tubule (rPT) cells that exposure to CdCl_2_ increases LC3-II and Beclin-1 expression, as well as the number of autophagic vacuoles, in a dose-dependent manner [51].

Nevertheless, the increase in LC3-II expression and in autophagosome numbers are not irrefutable proof of autophagy flux. The lack of lysosomal degradation evidence represents a more reliable parameter to confirm the autophagy flux [52], since autophagosome accumulation can derive from the increase in autophagosome formation or the suppression of lysosomal degradation [53]. In this regard it was shown that exposure to a subtoxic dose (0.3 mg Cd/kg) for 5 days did not affect tubular or glomerular function in rat kidneys, although CdCl_2_ accumulation in the renal cortex was observed [54]. However, proximal convoluted tubule cells (PCT)-exposure to a low dose of CdCl_2_, showed significant morphological changes associated to autophagy, but not to apoptosis. Furthermore, autophagy upregulation derived from the binding of CdCl_2_ to the sulfhydryl groups of proteins, oxidative stress, and the endoplasmic reticulum stress-dependent autophagy induction [54]. Similarly, Liu et al. (2017) showed in rPT cells exposed to CdCl_2_, the anti-apoptotic effect of autophagy with rapamycin treatment, which decreased the apoptosis rate by Fas/Fas ligand (FasL) pathway inhibition, in contrast to 3-methyladenine (3-MA) treatment, an autophagy inhibitor, which increased the apoptosis rate [51].

In support of this, Liu et al. [23] found in rPT cells treated with cadmium acetate (CdAc_2_) an increase in LC3-II and p62 protein expression, whose expression is inversely correlated with autophagic activity. This suggests autophagy flux inhibition, which was associated with reduction of the autophagosome-lysosome fusion, as a consequence of the cellular fusion machinery depletion and cytosolic calcium increase.

It is important to highlight that cadmium is not the only heavy metal able to impair autophagy flux. In vitro studies in rPT cells treated with lead nitrate (PbNO_3_) have shown the accumulation of LC3-II and p62 proteins [22,56]. Furthermore, autophagy inhibition increased the levels of cleaved caspase-3 and poly (ADP-ribose) polymerase (PARP), which evidenced its participation in lead-induced apoptosis in rPT cells [55]. The autophagy flux alterations in this condition were associated to autophagolysosome alkalinization, as a consequence of the suppression of the two V-ATPase subunits (which hydrolyze ATP to pump protons into the lysosome lumen). Furthermore, lead induced lysosomal membrane permeabilization (LMP), allowing cathepsins-release to the cytoplasm, which induced caspases-mediated apoptosis [22]. The authors also demonstrated the deregulation of the AMPK/mTOR pathway and suggested its participation in the lead-induced impairment of autophagy flux [56].

Autophagy flux disruption has also been observed in arsenic models. Kimura et al. (2016) found in female mice administered with sodium arsenite (NaAsO_2_), an accumulation of LC3-II and p62 proteins. The highlight of this study is that the participation of estrogens in the autophagic flux impairment was demonstrated, which affects more the females than the males. This phenomenon is associated with ERK overactivation, by SOCS3-dependent IL-6/STAT3 signaling pathway suppression [24].

About Cr (VI), its effect on autophagy in the kidneys is still unknown, but indirect evidence suggest that it may also inhibit the autophagy flux [58]. In rat kidney cortex and HK2 cells exposed to potassium dichromate (K_2_Cr_2_O_7_), there was an increase in p-mTOR and in phosphorylated-p70 ribosomal protein S6 kinase (p-p70S6K) (mTOR target) levels, which suggests the inhibition of autophagy flux [58].

## 5. Protective Effects of Curcumin Related to Autophagy Regulation

Curcumin can modulate autophagy flux by different molecular mechanisms. In addition, studies have proposed that curcumin acts in different ways depending on its concentration: At low concentrations it functions as an antioxidant, at medium concentrations it also acts as an autophagy inductor, however it has been reported in an in vitro experiment in cancer cell that curcumin at high concentrations leads to cell death [1,4,59,60]. Although the mechanisms by which curcumin induces autophagy were initially described in cancer cells [61], they also occur in normal cells [25]. Nevertheless, their effect differ between both cell types: In normal cells, curcumin mainly induces autophagy [62] in different ways to promote cell survival (see detail in Table 2). Meanwhile, in cancer cells, curcumin induces autophagy over-activation leading to cell death [63,64].

Interestingly, the effects of curcumin administration on autophagic flow in kidney damage models have recently begun to be studied. In kidney diseases, curcumin stimulates autophagy activation at early times, while in neurological and cardiovascular diseases it prevents autophagy activation [9,65]. The main signaling pathway described for autophagy induction by curcumin involves the suppression of the PI3K/Akt/mTOR pathway [66], as well as the stimulation of the AMPK and ERK1/2 pathways [32]. In this respect, Wei et al. (2017) found in a model of advanced glycation end-product (AGE)-induced renal toxicity, that curcumin prevented apoptosis through PI3K/AKT pathway-dependent autophagy activation. In addition, the pretreatment with 3-MA, an autophagy inhibitor, corroborated the protective role of curcumin. Since 3-MA induced autophagy suppression, it stimulated AGE-induced apoptosis [67]. Importantly, curcumin alone did not enhance basal levels of autophagy-related proteins. Likewise, Hu et al. (2018) showed in human kidney cells (HK-2) exposed to H_2_O_2_, an autophagy flux reduction related to oxidative stress increase [66].

Furthermore, curcumin administration protected from damage in this model, which is attributable to its ability to restore the autophagy flux by the inhibition/dephosphorylation of the Akt/mTOR pathway. On the other hand, Yao et al. (2018) found in in vitro and in vivo models of diabetic cardiomyopathy, that curcumin prevented apoptosis by restoring autophagy flux. Such process was mediated by JNK and AMPK/mTOR pathway activation. Furthermore, both kinases favored Bcl-2 phosphorylation, which disrupts its interaction with Beclin-1, an essential autophagy protein that initiates the autophagosome formation [68]. It is known that interaction of Beclin-1 with Bcl-2 does not allow the Beclin-1-binding to vacuolar protein sorting 34 (Vps34), meaning that Beclin-1-dependent autophagy is inhibited [69]. Stimulation of Beclin-1-dependent autophagy by curcumin has also been described in another injury type [70] as well as in various cancer cell lines [34].

In addition, curcumin not only participates in the early stage of autophagy, but also in the latter stage, such as in the fusion of autophagosomes and lysosomes, as well as in the degradation of autolysosomes [25,71]. Yan et al. (2018) found in mouse embryonic fibroblasts (MEF) cells exposed to curcumin, enhanced autophagic flux and lysosomal function (lysosomal acidification and cathepsin B activity) and an increased autophagosome-lysosome fusion. Lysosomal acidification by curcumin was achieved by upregulation of vacuolar V-ATPase gene expression [25]. Curcumin-induced autophagy activation was mediated by suppression of the AKT/mTOR pathway, although until now it is still under debate if the lysosomal function in autophagy depends on mTOR [25]. In support of this, Zhang et al. (2016) observed in Tsc^-/-^ MEF cells, in which mTOR is constitutively active, that curcumin was unable to enhance the lysosomal function, suggesting mTOR-dependent lysosomal function. Likewise, it also showed a direct interaction between curcumin and the transcription factor EB (TFEB), which is key for the control of autophagy and lysosomal biogenesis, in MEF and HCT116 cells, which was evidenced by the increase of lysosomal acidification and enhanced cathepsin B activity [71]. In this sense, mTOR-dependent phosphorylation of TFEB (S211) is recognized by YWHA/14-3-3 proteins that promote its cytoplasmic retention [72], but curcumin inhibits this phosphorylation, allowing TFEB translocation to the nucleus and enhancing its transcriptional activity [71].

Notably, curcumin is also effective in chronic kidney disease. A study in human kidney tubular epithelial cells (HKCs) found that curcumin exposure avoided the epithelial-to-mesenchymal transition through Akt/mTOR/p70S6K pathway inhibition [28]. In the same way, in the 5/6 nephrectomy-induced chronic kidney damage model, curcumin decreased the phosphorylation of mTOR and its targets (p70S6K and 4E-BP1) [73]. Although in both cases the study of autophagy was not the aim, the possible participation of this may be considered, since the Akt/mTOR pathway modulation is considered a classic mechanism for the regulation of autophagy [74,75]. However, more profound studies are necessary to provide clarification.

By contrast, other studies have also found that curcumin can inhibit autophagy in kidney damage, mainly in models of transition to chronic stage, where autophagy activation had harmful effects [30]. Li et al. (2019) described curcumin as an autophagy inhibitor in a model of hyperoxaluria-induced nephrolithiasis, which reduced the apoptosis rate. Furthermore, in a model using a contrast agent as a nephrotoxicity inductor, it was shown an increase in LC3-II expression in tubular epithelial cells, podocytes, mesangial cells, and macula dense. However, curcumin treatment decreased LC3-II levels and avoided autophagic cell death [17]. In support of this, in a maleate-induced nephrotoxicity model, it was found that curcumin’s protective effect was associated with decreased autophagy [31].

Taken together, these evidences suggest that the modulation of autophagy (stimulation or suppression) may be one of the mechanisms through which curcumin promotes cell survival. However, future studies of autophagy modulation by curcumin in heavy metals-induced autophagy flux impairment are still needed.

### Summary of Heavy Metals-Induced Autophagy Flux Impairment in Renal Damage

**Figure 1:**
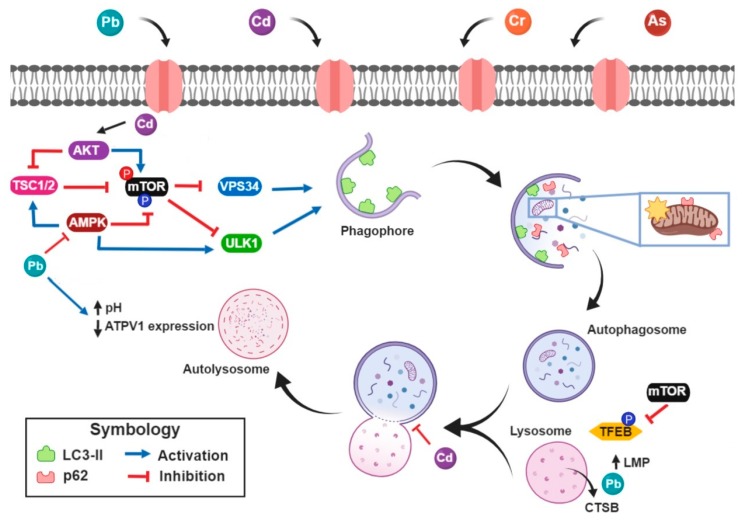
Integrative scheme. Heavy metals suppress the autophagy initiation step by the increase of the mammalian target of rapamycin (mTOR) activity derived from stimulation of protein kinase B (Akt) pathway or by adenosine monophosphate-activated protein kinase (AMPK) pathway inhibition. Furthermore, AMPK and Akt can enhance or reduce, respectively, the activity of tuberous sclerosis complex 1 (TSC1)/TSC2; which directly inhibits mTOR activity. In turn, multiprotein kinase complex 1 of mTOR promotes the phosphorylation/inactivation from Atg2/unc-51 like kinases complex (ULK) and vacuolar protein sorting 34 (VPS34), which are essential processes in the autophagy initiation. Furthermore, in the later stage, cadmium (Cd) and lead (Pb) impair autophagosome-lysosome fusion, elevate lysosomal pH at the expense of losing subunits of vacuolar-ATPase expression and allow cathepsin B (CTSB) release by lysosomal membrane permeabilization (LMP). Created with BioRender.com.

Heavy metals-induced impairment of autophagy flux occurs in both, the early stage and the later stage (as we summarized in Figure 1). In the autophagy initiation steps, the inhibition may be associated with the stimulation of the Akt pathway (e.g., Cd) and/or with the inhibition of the AMPK pathway (e.g., Pb). Meanwhile, at the later stage, heavy metals impair the autophagosome-lysosome fusion and/or avoid lysosomal acidification. These alterations are closely related to lysosomal function loss.

On the other hand, despite the lack of studies of curcumin’s effects on heavy metals-induced autophagy impairment, it has reported that curcumin modulates autophagy flux in many other models, by the inhibition of mTOR activity by Akt pathway suppression [25] or by AMPK pathway activation [68], as well as by the endorsement of lysosomal function (lysosomal acidification and cathepsin activity) probably through direct interaction with TFEB [71]. Although several studies support the idea that curcumin can restore autophagy flux alterations in several models, aspects such as cellular type, stimulus intensity, and curcumin concentration must be considered.

Finally, it has been reported in other renal damage models that curcumin’s nephroprotective effects are related to its ability to regulate autophagy flow [17,67]. However, future studies of autophagy modulation by curcumin in heavy metals-induced autophagy flux impairment are still needed.

## 6. Conclusions

Curcumin administration has demonstrated the ability to restore autophagy flux balance. Therefore, its administration in heavy metals-induced renal damage could be a possible treatment strategy to reverse the autophagy impairment, thus contributing to the preservation of renal function.

## Figures and Tables

**Table 1:** In vitro and in vivo effects of heavy metals on autophagy in kidney.

**In Vitro**			
**Cell Line and Treatment**	**Effect**	**Cause/Mechanism**	**References**
rPT cells CdCl_2_ (1.25–5 μM) for 12 h	↑ LC3-II and p62 protein ↓ Autophagosome-lysosome fusion ↑ Ca^2+^ cytosolic levels - Chelation (BAPTA-AM and 2-APB): ↑ Autophagosome-lysosome fusion ↑ Fusion machinery (Rab7 expression)	Ca^2+^ cytosolic overload impairment autophagic flux by autophagosome-lysosome fusion inhibition	[23]
HEK cells CdCl_2_ (0–80 μM) for 3, 6, and 12 h	↑ LC3-II, p-Akt and COX-2 protein ↓ p62 protein - COX-2 siRNA prevented: Changes in LC3-II, p-Akt, p62 and p-mTOR protein	COX-2 activated autophagy by Akt/mTOR inhibition	[48]
PCT cells CdCl_2_ (5 µM) for 1, 3, and 5 h	↑ LC3-II expression ↑ Ubiquitinated proteins - CdCl_2_ + inhibitors 3-MA: LC3-II non expression Baf: ↑↑LC3-II accumulation	Ubiquitinated proteins induced ER stress and autophagy activation as mechanisms to remove and detoxify the cell against CdCl_2_ toxicity	[54]
rPT cells CdCl_2_ (2.5–5 μM) for 6 and 12 h	↑ LC3-II and Beclin-1 protein - Interaction between beclin-1 and cleaved caspase-8 - Autophagy activation (rapamycin): ↓ Fas, FasL, FADD and cleaved caspase-8 protein, as well as apoptosis rate	Beclin-1 expression prevented Fas/FasL pathway-mediated apoptosis	[51]
rPT cells Pb(NO3)_2_ (0.5 µM) for 12 h	↑ LC3-II, p62, Beclin-1 and Atg5 proteins ↑ Autophagosome accumulation ↑ Apoptosis-related proteins (cleaved caspase-3 and cleaved PARP)	Autophagy inhibition is directly involved in Pb(NO3)_2_-induced apoptosis	[55]
rPT cells PbNO_3_ (0.5 μM) for 12 h	↑ LC3-II, p62, p-mTOR, p-p70S6K and p-4E-BP1 protein ↑ Autophagosome accumulation ↓ p-AMPK protein	Autophagic flux impairment by deregulation of AMPK/mTOR pathway	[56]
rPT cells PbAc_2_ (0.5 μM) for 12 h	↑ LC3-II and p62 protein ↑ Autophagosome accumulation ↓ Lysosomal alkalinization ↓ ATP6V1A and ATP6V1B1+ ATPV6VB2 protein subunits ↑ LMP and cathepsins release	Autophagic flux impairment by lysosomal alkalinization and LMP	[22]
rTP and NRK-52E cells CdAc_2_ (2.5–5 μM) for 12 h	↑ LC3-II, Beclin-1 and Atg5 proteins ↑ Autophagosome accumulation ↓ p62 protein ↑ p-ERK1/2 stimulated autophagy ↑ p-JNK1/2 and p-38 promoted apoptosis - ERK inhibition (U0126): ↑ ER stress-induced apoptosis ↓ Autophagy activation	p-ERK1/2-induced autophagy activation can suppress ER stress-mediated apoptosis	[57]
**In Vivo**			
Male kunming mice CdCl_2_ (0.4 mg/kg/day, i.p.) for 3 days	↑ LC3-II/LC3-I ratio ↑ Autophagosome formation	ROS-mediated autophagy	[49]
Male ICR mice CdCl_2_ (0.2 mg to 5 mg/kg/day, i.p.) for 1 week	↑ LC3-II and COX-2 protein ↓ p62 protein - COX-2 inhibition (celecoxib) prevented: Changes in LC3-II and p62 protein	COX-2 regulates CdCl_2_-induced autophagy	[48]
Female BALB/c mice NaAsO_2_ (12.5 mg/kg, s.c.)	↑ LC3-II/LC3-I, p62, SOCS3 and p-ERK/ERK protein ↑ SOCS3 mRNA, but IL-6 was unchanged ↓ p-STAT3/STAT3 protein	Impairment of autophagy flux resulted from ERK activation, by SOCS3 dependent-attenuation of IL-6/STAT3 activation	[24]
Female Wistar rats CdCl_2_ (0.3 mg/kg, i.p.) for 5 days	Glomerular and tubular functions not affected CdCl_2_ accumulation on renal cortex Structural changes in PCT cells associated to proliferation and autophagy	CdCl_2_ accumulation generated ubiquitinated proteins, in order to remove them, autophagy was activated	[54]
Hyline Brown laying chicken treated with CdCl_2_ (150 mg/kg in diet supplemented) for 90 days	↑ Autophagic vesicles ↑ JNK, Beclin-1, Atg5, LC3-I and LC3-II protein and mRNA levels	CdCl_2_ promotes JNK-dependent autophagy	[50]

2-APB: 2-aminoethoxidiphenyl borate; 3-MA: 3-methyladenine; 4E-BP1: 4E-binding protein; Akt: protein kinase B; AMPK: adenosine monophosphate-activated protein kinase; Atg5: autophagy-related gene 5; ATP6V1A: vacuolar-ATPase subunit A; ATP6V1B1: vacuolar-ATPase subunit B1; ATP6V1B2: vacuolar-ATPase subunit B2; Baf: bafilomycin; Bax: Bcl-2-associated X protein; Bcl-2: B-cell lymphoma; COX-2: cyclooxygenase-2; ER: endoplasmic reticulum; ERK: extracellular signal-regulated kinase 1/2; FADD: Fas-associated death domain-containing protein; FasL: Fas ligand; IL-6: interleukin-6; JNK: c-Jun N-terminal kinase 1/2; LC3-I: microtubule-associated protein 1 light chain 3; LC3-II: LC3 conjugated with phosphatidylethanolamine; LMP: lysosomal membrane permeabilization; mTOR: mammalian target of rapamycin; p62: ubiquitin-binding protein p62; p70S6K: p70 ribosomal protein S6 kinase; PARP: poly (ADP-ribose) polymerase; PCT: proximal convoluted tubule; PI3K: phosphatidylinositol 3-kinase; rPT: rat proximal tubule; SOCS3: suppressor of cytokine signaling 3; STAT3: signal transducer and activator of transcription 3.

**Table 2:** Curcumin effects on autophagy.

Experimental Model	Conditions	Curcumin Effect	References
HKC cells	Cells were treated with TGF-β1 (5 ng/mL) alone or combination with curcumin (3.125–25 μM) for 72 h	↓ Vimentin and α-SMA mRNA and protein levels ↓ p-mTOR, p-Akt, p-p70S6K, and p-4E-BP1 protein ↓ Epithelial-to-mesenchymal transition	[28]
MEF cells	20 μM curcumin for 12 h	↑ LC3-II and ↓ p62 protein ↑ Lysosomal acidification, enzyme activity of lysosomal cathepsin B and autophagosome-lysosome fusion ↑ Atp6v1a, Atp6voc, and Atp6voe mRNA levels ↓ p-Akt, p-mTOR and p70S6K protein	[25]
MEF and HTC116 cells	20 μM curcumin for 12 h	↑ LC3-II protein ↓ p62, p-Akt, p-mTOR, p70S6K, and p-TFEB protein ↑ Lysosomal acidification and enzyme activities of lysosomal cathepsin B ↑ Tfeb, Lamp1, and Atp6v1a mRNA levels ↑ TFEB translocation and transcriptional activity In Tsc2^-/-^ MEFs: Curcumin was unable to increase the lysosomal function	[71]
Wistar-albino rats	Curcumin for 10 days (200 mg/kg/day, oral). On the 5th day contrast agent was administered	↓ Kidney damage markers (Scr, BUN) ↓ Lipid peroxidation ↑ Antioxidant activity (SOD, CAT) ↓ LC3-II expression in tubular epithelial cells, podocytes and mesangial cells in the glomeruli and the macula dense ↓ Caspase-3 activation	[17]
NRK-52E cells	Co-incubation of AGE (700 μg/mL) with curcumin (10 μM) for 48 h	↓ Apoptosis-related proteins (Bax, AIF and caspase-3) ↑ LC3-II and Beclin-1 and p-Akt levels 3-MA pretreatment: - Suppression of curcumin-induced apoptosis inhibition LY294002 (PI3K/AKT inhibitor) pretreatment: - Avoided the curcumin-induced autophagy activation	[67]
Male C57BL/6 mice and H9c2 cells	STZ-induced diabetic mice were treated with curcumin (200 mg/kg/day in drinking water) for 3 months Cells were co-incubated at high concentrations of D-glucose (30 mM) plus palmitate (0.1 mM) with curcumin for 24 to 36 h.	In both: ↓ Apoptosis-related proteins (cytochrome c and caspase-3 cleaved) ↑ Autophagic-like vesicle formation ↑ p-AMPK, p-JNK, p-Bcl-2, and Bim protein levels ↓ p-mTOR, p-p70S6K, and p-4E-BP1 protein levels In vitro: ↓ Interaction of Beclin1 with Bcl-2 and Bim 3-MA pretreatment: - Suppressed autophagic flux and increase apoptosis rate Inhibition of JNK- and AMPK-activation: - Abolished ability of curcumin to enhance LC3-II formation and to suppress apoptosis	[68]
Tubular epithelial (HK-2) cells and ICR male mice	Cells pretreated with H_2_O_2_ (200 μM) for 4 h followed by curcumin (20 μg/mL) incubation for 12 h Curcumin (10 mg/kg, i.v.) was administered to animals in an ischemia (30 min)/reperfusion (24 h) model	In vitro: ↓ Apoptosis (caspase-3 and -9) ↑ Autophagic-like vesicle formation and LC3-II protein ↓ p62, p-Akt and p-mTOR protein In vivo: ↓ Kidney damage markers (Scr, BUN) ↓ Tubular necrosis, luminal congestion, and pro-inflammatory cytokine (TNF-α and IL-6)	[66]
Male Sprague-Dawley rats	Rats that underwent 5/6 nephrectomy were administered with curcumin (75 mg/day, oral) for 6 weeks.	↓ Kidney damage markers (Scr, BUN and proteinuria) ↓ Glomerular hypertrophy, tubular dilation, and fibrosis ↓ mTOR, p-mTOR, p-p70S6K, and p-4E-BP1	[73]

4E-BP1: 4E-binding protein; 3-MA: 3-methyladenine; AGE: advanced glycation end-product; AIF: apoptosis-inducing factor; Akt: protein kinase B; AMPK: adenosine monophosphate-activated protein kinase; α-SMA: alpha smooth muscle actin; Bax: Bcl-2-associated X protein; Bcl-2: B-cell lymphoma; BUN: blood urea nitrogen; CAT: catalase; HKC: human kidney tubular epithelial cells; IL-6: interleukin-6; JNK: c-Jun N-terminal kinase; LC3-I: microtubule-associated protein 1 light chain 3; LC3-II: LC3 conjugated with phosphatidylethanolamine; mTOR: mammalian target of rapamycin; p62:ubiquitin-binding protein p62, p70S6K: p70 ribosomal protein S6 kinase; Scr: serum creatinine; SOD: superoxide dismutase; STZ: streptozotocin; TFEB: transcription factor EB; TGF-β1: transforming growth factor betta; TNF-α: tumor necrosis factor alpha; TSC: tuberous sclerosis complex.

## References

[1] Moghaddam N.S.A., Oskouie M.N., Butler A.E., Petit P.X., Barreto G.E., Sahebkar A. (2019). Hormetic effects of curcumin: What is the evidence?. J. Cell. Physiol..

[2] Kocaadam B., Şanlier N. (2017). Curcumin, an active component of turmeric (*Curcuma longa*), and its effects on health, Crit. Rev. Food Sci. Nutr..

[3] de Oliveira M.R., Jardim F.R., Setzer W.N., Nabavi S.M., Nabavi S.F. (2016). Curcumin, mitochondrial biogenesis, and mitophagy: Exploring recent data and indicating future needs. Biotechnol. Adv..

[4] Rainey N., Motte L., Aggarwal B.B., Petit P.X. (2015). Curcumin hormesis mediates a cross-talk between autophagy and cell death. Cell Death Dis..

[5] García-Niño W.R., Pedraza-Chaverrí J. (2014). Protective effect of curcumin against heavy metals-induced liver damage. Food Chem. Toxicol..

[6] Liu F., Gao S., Yang Y., Zhao X., Fan Y., Ma W., Yang D., Yang A., Yu Y. (2018). Antitumor activity of curcumin by modulation of apoptosis and autophagy in human lung cancer A549 cells through inhibiting PI3K/Akt/mTOR pathway. Oncol. Rep..

[7] Mounce B.C., Cesaro T., Carrau L., Vallet T., Vignuzzi M. (2017). Curcumin inhibits Zika and chikungunya virus infection by inhibiting cell binding. Antivir. Res..

[8] Zhang J., Tang L., Li G.S., Wang J. (2018). The anti-inflammatory effects of curcumin on renal ischemia-reperfusion injury in rats. Ren. Fail..

[9] Huang L., Chen C., Zhang X., Li X., Chen Z., Yang C., Liang X., Zhu G., Xu Z. (2018). Neuroprotective Effect of Curcumin Against Cerebral Ischemia-Reperfusion Via Mediating Autophagy and Inflammation. J. Mol. Neurosci..

[10] Jin T., Song Z., Weng J., Fantus I.G. (2018). Curcumin and other dietary polyphenols: Potential mechanisms of metabolic actions and therapy for diabetes and obesity. Am. J. Physiol. Metab..

[11] Patel S.S., Acharya A., Ray R.S., Agrawal R., Raghuwanshi R., Jain P. (2019). Cellular and molecular mechanisms of curcumin in prevention and treatment of disease. Crit. Rev. Food Sci. Nutr..

[12] Avila-Rojas S.H., Tapia E., Briones-Herrera A., Aparicio-Trejo O.E., León-Contreras J.C., Hernández-Pando R., Pedraza-Chaverri J. (2018). Curcumin prevents potassium dichromate (K2Cr2O7)-induced renal hypoxia. Food Chem. Toxicol..

[13] Lao C.D., Ruffin M.T., Normolle D., Heath D.D., Murray S.I., Bailey J.M., Boggs M.E., Crowell J., Rock C.L., Brenner D.E. (2006). Dose escalation of a curcuminoid formulation. BMC Complement. Altern. Med..

[14] Kim K.S., Lim H.J., Lim J.S., Son J.Y., Lee J., Lee B.M., Chang S.C., Kim H.S. (2018). Curcumin ameliorates cadmium-induced nephrotoxicity in Sprague-Dawley rats. Food Chem. Toxicol..

[15] Soliman M.M., Baiomy A.A., Yassin M.H. (2015). Molecular and Histopathological Study on the Ameliorative Effects of Curcumin Against Lead Acetate-Induced Hepatotoxicity and Nephrototoxicity in Wistar Rats. Biol. Trace Elem. Res..

[16] Aparicio-Trejo O.E., Tapia E., Molina-Jijón E., Medina-Campos O.N., Macías-Ruvalcaba N.A., León-Contreras J.C., Hernández-Pando R., García-Arroyo F.E., Cristóbal M., Sánchez-Lozada L.G. (2017). Curcumin prevents mitochondrial dynamics disturbances in early 5/6 nephrectomy: Relation to oxidative stress and mitochondrial bioenergetics. Biofactors.

[17] Buyuklu M., Kandemir F.M., Ozkaraca M., Set T., Bakirci E.M., Topal E. (2014). Protective effect of curcumin against contrast induced nephropathy in rat kidney: What is happening to oxidative stress, inflammation, autophagy and apoptosis?. Eur. Rev. Med. Pharmacol. Sci..

[18] Rana M.N., Tangpong J., Rahman M.M. (2018). Toxicodynamics of Lead, Cadmium, Mercury and Arsenic- induced kidney toxicity and treatment strategy: A mini review. Toxicol. Rep..

[19] Lentini P., Zanoli L., Granata A., Signorelli S.S., Castellino P., Dellaquila R. (2017). Kidney and heavy metals - The role of environmental exposure. Mol. Med. Rep..

[20] Wu W., Zhang K., Jiang S., Liu D., Zhou H., Zhong R., Zeng Q., Cheng L., Miao X., Tong Y. (2018). Association of co-exposure to heavy metals with renal function in a hypertensive population. Environ. Int..

[21] Abdel-Moneim A.M., El-Toweissy M.Y., Ali A.M., Allah A.A.M.A., Darwish H.S., Sadek I.A. (2015). Curcumin Ameliorates Lead (Pb2+)-Induced Hemato-Biochemical Alterations and Renal Oxidative Damage in a Rat Model. Biol. Trace Elem. Res..

[22] Song X.B., Liu G., Liu F., Yan Z.G., Wang Z.Y., Liu Z.P., Wang L. (2017). Autophagy blockade and lysosomal membrane permeabilization contribute to lead-induced nephrotoxicity in primary rat proximal tubular cells. Cell Death Dis..

[23] Liu F., Wang X.Y., Zhou X.P., Liu Z.P., Song X.B., Wang Z.Y., Wang L. (2017). Cadmium disrupts autophagic flux by inhibiting cytosolic Ca 2+ -dependent autophagosome-lysosome fusion in primary rat proximal tubular cells. Toxicology.

[24] Kimura A., Ishida Y., Nosaka M., Kuninaka Y., Hama M., Kawaguchi T., Sakamoto S., Shinozaki K., Iwahashi Y., Takayasu T. (2016). Exaggerated arsenic nephrotoxicity in female mice through estrogen-dependent impairments in the autophagic flux. Toxicology.

[25] Yan P., Sun X., Chen X., Chen Y., Wang X., Su D., Zhou H., Gao L., Lu L., Wang J. (2018). The Polyphenolic Compound Curcumin Conjugation with an Alkyne Moiety in the Process of Autophagy. Am. J. Chin. Med..

[26] Tan J., Wang M., Song S., Miao Y., Zhang Q. (2018). Autophagy activation promotes removal of damaged mitochondria and protects against renal tubular injury induced by albumin overload. Histol. Histopathol..

[27] Yang X., Yan X., Yang D., Zhou J., Song J., Yang D. (2018). Rapamycin attenuates mitochondrial injury and renal tubular cell apoptosis in experimental contrast-induced acute kidney injury in rats. Biosci. Rep..

[28] Zhu F., Chen M., Zhu M., Zhao R., Qiu W., Xu X., Liu H., Zhao H., Yu R., Wu X. (2017). Curcumin Suppresses Epithelial—Mesenchymal Transition of Renal Tubular Epithelial Cells through the Inhibition of Akt/mTOR Pathway. Biol. Pharm. Bull..

[29] Molina-Jijón E., Tapia E., Zazueta C., el Hafidi M., Zatarain-Barrón Z.L., Hernández-Pando R., Medina-Campos O.N., Zarco-Márquez G., Torres I., Pedraza-Chaverri J. (2011). Curcumin prevents Cr(VI)-induced renal oxidant damage by a mitochondrial pathway, Free Radic. Biol. Med..

[30] Li Y., Zhang J., Liu H., Yuan J., Yin Y., Wang T., Cheng B., Sun S., Guo Z. (2019). Curcumin ameliorates glyoxylate-induced calcium oxalate deposition and renal injuries in mice. Phytomedicine.

[31] Molina-Jijón E., Aparicio-Trejo O.E., Rodríguez-Muñoz R., León-Contreras J.C., Cárdenas-Aguayo M.d., Medina-Campos O.N., Tapia E., Sánchez-Lozada L.G., Hernández-Pando R., Reyes J.L. (2016). The nephroprotection exerted by curcumin in maleate-induced renal damage is associated with decreased mitochondrial fission and autophagy. BioFactors.

[32] Shakeri A., Cicero A.F.G., Panahi Y., Mohajeri M., Sahebkar A. (2019). Curcumin: A naturally occurring autophagy modulator. J. Cell. Physiol..

[33] Trujillo J., Granados-Castro L.F., Zazueta C., Andérica-Romero A.C., Chirino Y.I., Pedraza-Chaverrí J. (2014). Mitochondria as a Target in the Therapeutic Properties of Curcumin. Arch. Pharm..

[34] Zenkov N.K., Chechushkov A.V., Kozhin P.M., Kandalintseva N.V., Martinovich G.G., Menshchikova E.B. (2016). Plant phenols and autophagy. Biochemistry.

[35] Ortega-Domínguez B., Aparicio-Trejo O.E., García-Arroyo F.E., León-Contreras J.C., Tapia E., Molina-Jijón E., Hernández-Pando R., Sánchez-Lozada L.G., Barrera-Oviedo D., Pedraza-Chaverri J. (2017). Curcumin prevents cisplatin-induced renal alterations in mitochondrial bioenergetics and dynamic. Food Chem. Toxicol..

[36] Gaedeke J., Noble N.A., Border W.A. (2005). Curcumin blocks fibrosis in anti-Thy 1 glomerulonephritis through up-regulation of heme oxygenase 1. Kidney Int..

[37] Ghosh S.S., Massey H.D., Krieg R., Fazelbhoy Z.A., Ghosh S., Sica D.A., Fakhry I., Gehr T.W.B. (2009). Curcumin ameliorates renal failure in 5/6 nephrectomized rats: Role of inflammation. Am. J. Physiol..

[38] Liu F.H., Ni W.J., Wang G.K., Zhang J.J. (2016). Protective role of curcumin on renal ischemia reperfusion injury via attenuating the inflammatory mediators and Caspase-3. Cell. Mol. Biol..

[39] Guerrero-Hue M., García-Caballero C., Palomino-Antolín A., Rubio-Navarro A., Vázquez-Carballo C., Herencia C., Martín-Sanchez D., Farré-Alins V., Egea J., Cannata P. (2019). Curcumin reduces renal damage associated with rhabdomyolysis by decreasing ferroptosis-mediated cell death. FASEB J..

[40] Pickles S., Vigié P., Youle R.J. (2018). Mitophagy and Quality Control Mechanisms in Mitochondrial Maintenance. Curr. Biol..

[41] Sureshbabu A., Ryter S.W., Choi M.E. (2015). Oxidative stress and autophagy: Crucial modulators of kidney injury. Redox Biol..

[42] Hashemzaei M., Heravi R.E., Rezaee R., Roohbakhsh A., Karimi G. (2017). Regulation of autophagy by some natural products as a potential therapeutic strategy for cardiovascular disorders. Eur. J. Pharmacol..

[43] Kaushal G.P., Shah S.V. (2016). Autophagy in acute kidney injury. Kidney Int..

[44] Dikic I., Elazar Z. (2018). Mechanism and medical implications of mammalian autophagy. Nat. Rev. Mol. Cell Biol..

[45] Navarro-Yepes J., Burns M., Anandhan A., Khalimonchuk O., del Razo L.M., Quintanilla-Vega B., Pappa A., Panayiotidis M.I., Franco R. (2014). Oxidative stress, redox signaling, and autophagy: Cell death versus survival. Antioxid. Redox Signal..

[46] Wang Z., Choi M.E. (2014). Autophagy in Kidney Health and Disease. Antioxid. Redox Signal..

[47] Lin F. (2017). Autophagy in renal tubular injury and repair. Acta Physiol..

[48] Luo B., Lin Y., Jiang S., Huang L., Yao H., Zhuang Q., Zhao R., Liu H., He C., Lin Z. (2016). Endoplasmic reticulum stress eIF2α–ATF4 pathway-mediated cyclooxygenase-2 induction regulates cadmium-induced autophagy in kidney. Cell Death Dis..

[49] Yuan Y., Ma S., Qi Y., Wei X., Cai H., Dong L., Lu Y., Zhang Y., Guo Q. (2016). Quercetin inhibited cadmium-induced autophagy in the mouse kidney via inhibition of oxidative stress. J. Toxicol. Pathol..

[50] Shi Q., Jin X., Fan R., Xing M., Guo J., Zhang Z., Zhang J., Xu S. (2019). Cadmium-mediated miR-30a-GRP78 leads to JNK-dependent autophagy in chicken kidney. Chemosphere.

[51] Liu G., Yuan Y., Long M., Luo T., Bian J., Liu X., Gu J., Zou H., Song R., Wang Y. (2017). Beclin-1-mediated Autophagy Protects Against Cadmium-activated Apoptosis via the Fas/FasL Pathway in Primary Rat Proximal Tubular Cell Culture. Sci. Rep..

[52] Jiang P., Mizushima N. (2015). LC3- and p62-based biochemical methods for the analysis of autophagy progression in mammalian cells. Methods.

[53] Ni H.M., Bockus A., Wozniak A.L., Jones K., Weinman S., Yin X.M., Ding W.X. (2011). Dissecting the dynamic turnover of GFP-LC3 in the autolysosome. Autophagy.

[54] Chargui A., Zekri S., Jacquillet G., Rubera I., Ilie M., Belaid A., Duranton C., Tauc M., Hofman P., Poujeol P. (2011). Cadmium-induced autophagy in rat kidney: An early biomarker of subtoxic exposure. Toxicol. Sci..

[55] Chu B.X., Fan R.F., Lin S.Q., Yang D.B., Wang Z.Y., Wang L. (2018). Interplay between autophagy and apoptosis in lead(II)-induced cytotoxicity of primary rat proximal tubular cells. J. Inorg. Biochem..

[56] Song X., Li Z., Liu F., Wang Z., Wang L. (2017). Restoration of autophagy by puerarin in lead-exposed primary rat proximal tubular cells via regulating AMPK-mTOR signaling. J. Biochem. Mol. Toxicol..

[57] Luo T., Zhang H., Yu Q., Liu G., Long M., Zhang K., Liu W., Song R., Bian J., Gu J. (2018). ERK1/2 MAPK promotes autophagy to suppress ER stress-mediated apoptosis induced by cadmium in rat proximal tubular cells. Toxicol. Vitr..

[58] Song M.F., Yang Y., Yi Z.W., Zhang Z.Q., Shen X.D., Hu G.H., Zhu Y.F. (2018). Sema 3A as a biomarker of the activated mTOR pathway during hexavalent chromium-induced acute kidney injury. Toxicol. Lett..

[59] Chen P., Huang H.P., Wang Y., Jin J., Long W.G., Chen K., Zhao X.H., Chen C.G., Li J. (2019). Curcumin overcome primary gefitinib resistance in non-small-cell lung cancer cells through inducing autophagy-related cell death. J. Exp. Clin. Cancer Res..

[60] Hsiao Y.T., Kuo C.L., Chueh F.S., Liu K.C., Bau D.T., Chung J.G. (2018). Curcuminoids Induce Reactive Oxygen Species and Autophagy to Enhance Apoptosis in Human Oral Cancer Cells. Am. J. Chin. Med..

[61] Aoki H., Takada Y., Kondo S., Sawaya R., Aggarwal B.B., Kondo Y. (2007). Evidence That Curcumin Suppresses the Growth of Malignant Gliomas in Vitro and in Vivo through Induction of Autophagy: Role of Akt and Extracellular Signal-Regulated Kinase Signaling Pathways. Mol. Pharmacol..

[62] Gao Y., Li J., Wu L., Zhou C., Wang Q., Li X., Zhou M., Wang H. (2016). Tetrahydrocurcumin provides neuroprotection in rats after traumatic brain injury: Autophagy and the PI3K/AKT pathways as a potential mechanism. J. Surg. Res..

[63] Maiti P., Scott J., Sengupta D., Al-Gharaibeh A., Dunbar G. (2019). Curcumin and Solid Lipid Curcumin Particles Induce Autophagy, but Inhibit Mitophagy and the PI3K-Akt/mTOR Pathway in Cultured Glioblastoma Cells. Int. J. Mol. Sci..

[64] Zhu J., Zhao B., Xiong P., Wang C., Zhang J., Tian X., Huang Y. (2018). Curcumin Induces Autophagy via Inhibition of Yes-Associated Protein (YAP) in Human Colon Cancer Cells. Med. Sci. Monit..

[65] Liu R., Zhang H.B., Yang J., Wang J.R., Liu J.X., Li C.L. (2018). Curcumin alleviates isoproterenol-induced cardiac hypertrophy and fibrosis through inhibition of autophagy and activation of mTOR. Eur. Rev. Med. Pharmacol. Sci..

[66] Hu J.B., Li S.J., Kang X.Q., Qi J., Wu J.H., Wang X.J., Xu X.L., Ying X.Y., Jiang S.P., You J. (2018). CD44-targeted hyaluronic acid-curcumin prodrug protects renal tubular epithelial cell survival from oxidative stress damage. Carbohydr. Polym..

[67] Wei Y., Gao J., Qin L., Xu Y., Shi H., Qu L., Liu Y., Xu T., Liu T. (2017). Curcumin suppresses AGEs induced apoptosis in tubular epithelial cells via protective autophagy. Exp. Ther. Med..

[68] Yao Q., Ke Z.Q., Guo S., Yang X.S., Zhang F.X., Liu X.F., Chen X., Chen H.G., Ke H.Y., Liu C. (2018). Curcumin protects against diabetic cardiomyopathy by promoting autophagy and alleviating apoptosis. J. Mol. Cell. Cardiol..

[69] Mukhopadhyay S., Panda P.K., Sinha N., Das D.N., Bhutia S.K. (2014). Autophagy and apoptosis: Where do they meet?. Apoptosis.

[70] Li G., Chen L., Chen K. (2018). Curcumin Promotes Femoral Fracture Healing in a Rat Model by Activation of Autophagy. Med. Sci. Monit..

[71] Zhang J., Wang J., Xu J., Lu Y., Jiang J., Wang L., Shen H.M., Xia D. (2016). Curcumin targets the TFEB-lysosome pathway for induction of autophagy. Oncotarget.

[72] Song J.X., Sun Y.R., Peluso I., Zeng Y., Yu X., Lu J.H., Xu Z., Wang M.Z., Liu L.F., Huang Y.Y. (2016). A novel curcumin analog binds to and activates TFEB in vitro and in vivo independent of MTOR inhibition. Autophagy.

[73] He Y., Lang X., Cheng D., Yang Z. (2019). Curcumin ameliorates chronic renal failure in 5/6 nephrectomized rats by regulation of the mTOR/HIF-1α/VEGF signaling pathway. Biol. Pharm. Bull..

[74] Vergadi E., Ieronymaki E., Lyroni K., Vaporidi K., Tsatsanis C. (2017). Akt Signaling Pathway in Macrophage Activation and M1/M2 Polarization. J. Immunol..

[75] Heras-Sandoval D., Pérez-Rojas J.M., Hernández-Damián J., Pedraza-Chaverri J. (2014). The role of PI3K/AKT/mTOR pathway in the modulation of autophagy and the clearance of protein aggregates in neurodegeneration. Cell Signal..

